# Extramural gerontology management devising an integrating record for a geriatric care service, an experience report

**DOI:** 10.1590/1980-57642021dn15-010012

**Published:** 2021

**Authors:** Thais Bento Lima-Silva, Evany Bettine de Almeida, Felipe Souza Peito Silva Borges, Tiago Nascimento Ordonez, Marisa Accioly Rodrigues Domingues

**Affiliations:** 1 Escola de Artes, Ciências e Humanidades, Universidade de São Paulo – São Paulo, SP, Brazil.

**Keywords:** aging, older adults, comprehensive health care for the elderly, health services management, gerontology, envelhecimento, pessoa idosa, atenção integral à, saúde do idoso, gestão dos serviços de saúde, gerontologia

## Abstract

The Gerontological Care Plan is idealized through case management that includes in its aspect engaging the elderly, self-care and the acquisition and maintenance of health-promoting behaviors.

**Objective:**

To evaluate the importance of a gerontological care plan, in a geriatric service of a referral hospital in the city of São Paulo.

**Methods:**

Fifteen older adult patients were interviewed and the Gerontological Care Plan (PAGe) was applied.

**Results:**

Most respondents were classified as independent for instrumental activities of daily living, 42% of whom lived alone. Data from 277 yellow sheets were analyzed, that is, referral forms, in which it was found that the most affected areas were: social work and psychology. For the social worker, the most recurring requests were: verification of the social support network, namely lack of companion and caregiver, with 53%; family problems, with 20%; lack of adherence to treatment, 12%, and problems related to medication, 10%. In the area of psychology, 82% of referrals were due to the need for psychological support, psychotherapy, and help with family problems, depression and grief.

**Conclusions:**

A gerontological management proposal was developed within the Geriatric Services of Hospital das Clínicas. The management plan was intended to integrate the actions carried out by the interprofessional team, through the creation of an Integrating Form that allowed the gerontologist to propose, execute and implement a plan of care, follow-up, and monitoring of cases, including the extra context-hospital.

## RESUMO.

O Plano de Atenção Gerontológica é idealizado por meio da gestão de casos que engloba em sua vertente, engajamento da pessoa idosa, autocuidado e aquisição e manutenção de comportamentos de promoção de saúde.

**Objetivo:**

Avaliar a importância de um plano de atenção gerontológica em um serviço de geriatria de um hospital de referência na cidade de São Paulo.

**Métodos:**

Foram entrevistados 15 pacientes idosos, sendo aplicado o Plano de Atenção Gerontológica (PAGe).

**Resultados:**

Em sua maioria, os entrevistados foram classificados como independentes para as atividades instrumentais de vida diária, sendo que 42% deles residem sozinhos. Dados de 277 folhas amarelas foram analisados, ou seja, Fichas de Encaminhamento, nos quais foi possível verificar que as áreas mais acionadas foram o serviço social e a psicologia. Para assistência social, as solicitações mais recorrentes foram a verificação da rede de suporte social, falta de acompanhante e de cuidador, com 53%, problemas familiares, com 20%, falta de adesão ao tratamento, 12%, e problemas relacionados a medicamentos, 10%. Na área da psicologia, 82% dos encaminhamentos foram feitos por necessidade de acompanhamento psicológico/psicoterapia, problemas com família, depressão e luto.

**Conclusões:**

Elaborou-se uma proposta de Gestão Gerontológica dentro dos Serviços de Geriatria do Hospital das Clínicas. O plano de gestão teve o intuito de integrar as ações realizadas pela equipe interprofissional, por meio da criação de uma Ficha Integradora que permitiu ao profissional gerontólogo propor, executar e implementar um plano de atenção, acompanhamento e monitoramento dos casos, incluindo o contexto extra-hospitalar.

## INTRODUCTION

In the past 20 years, Brazil’s ranking among countries with the largest elderly populations rose from 16^th^ to 10^th^ in the world. By 2025, the proportion of the nation’s elderly population is set to reach 15.1%, making Brazil a country of elderly as opposed to younger individuals.^1^

This rise in the number of elderly, from a health perspective, translates to an increase in long-term chronic conditions and diseases, which often require high-cost interventions involving complex technology for adequate treatment. Consequently, the aging population poses a socioeconomic challenge for the government and society.^2^ Thus, short-, medium- and long-term planning is vital to establish a social well-being and healthcare policy for Brazil’s elderly population, involving a multidisciplinary team and professionals specialized in gerontology.

The elderly should be evaluated globally, and parameters such as functional capacity and cognitive, social, psychological and cultural aspects should never be left to the background, when thinking about doing case management with the performance of the interdisciplinary team in health institutions.^3^ ^,^ ^4^

Protocols do not always allow the fulfillment of these requirements; however, currently, knowledge and studies demonstrate the effectiveness of using a Comprehensive Gerontological Assessment. In this sense, one of the instruments of knowledge of the gerontologist, such as the Gerontological Assessment (PAGe) that proposes integrated and shared actions, seeks to meet the aforementioned requirements.^1^ ^,^ ^2^

As well as being fundamental tools for medium- and long-term therapeutic planning, it is an essential part of the inter- and multidisciplinary approach to the management of care for the elderly. It should be noted that the multidimensional assessment instruments for the older adults, as previously reported, are essential in the management of the individual’s routine and care.^2^

Accordingly, it is not possible to dissociate case management from broad gerontological assessment, as this assessment, which is one of the gerontologist’s screening tools, is also associated with the concept of promoting healthy aging, by tracking physical, cognitive, functional changes, having as goals the stimulation, rehabilitation and/or functional preservation, through a performance with an interdisciplinary team. Thus, contributing to the promotion of longevity, and increase in life expectancy with health, autonomy and independence, thus reducing risks of institutionalization, hospitalizations and recurrent hospitalizations.^3^ ^,^ ^4^

On the basis of this context, our objective was to evaluate the importance of a gerontological care plan, in a geriatric service of a referral hospital in the city of São Paulo. Also, we aimed to carry out a documentary analysis of medical records, through retrospective analysis, and to fill out an integrating form of practices performed by geriatric services of the same institution, to optimize the communication between the institution’s services and the management plan carried out. by the interdisciplinary team that was caring for elderly users, to have humanized and integrated referrals.

## METHODS

### Study type

A descriptive exploratory study of quantitative data based on information collected at each of the different areas of medical residency was conducted. The study was performed in 3 sectors of the Geriatrics Service of Hospital das Clinicas: the Infirmary, Outpatient Clinic and Day-Hospital. Patients and health professionals from the 3 sectors took part in the study.

### Participants and protocol

The following instruments were used: the Gerontology Care Plan (PAGe) encompassing a battery of instruments for biopsychosocial assessment of elderly, an analysis of patient referrals to professionals at Hospital das Clínicas (via Yellow Sheets), and an interview with professionals heading each area of residency. The PAGe instrument was developed by the instructors in the gerontology degree program of the Universidade São Paulo, on the basis of the gerontology assessment instruments recommended in the Handbook on Primary Health for the Elderly of the Ministry of Health^5^. The objective of PAGe is to collect information on biopsychosocial aspects of older adults for integrated assessment. These results then provide a basis for devising a management plan. The instrument contains questions on habits, nutrition (outside hospital), functional autonomy, sleep, spirituality, falls, depression, risk screening, disease besides physical, and psychological, socioeconomic and family status.^6^

PAGe also has a field for the gerontologist’s assessment and for suggested intervention to resolve problems identified, i.e., to devise a management plan together with the elderly patient/caregiver. Lastly, there is a section for a management plan that recruits other professionals on the team.

To obtain a broader picture of the life of the elderly and to better cater to their needs, PAGe can be used in conjunction with other instruments, such as: scales assessing Activities of Daily Living (ADLs) and Instrumental Activities of Daily Living (IADLs), Geriatric Depression Scale (GDS), Functionality Scale, Cognitive Screening Scale, Measures of Subjective and Psychological Well-being, Beliefs and Attitudes about Aging and others.

A total of 15 elderly patients were interviewed using the PAGe, applied between March and May 2010 at the different areas of residency within Hospital das Clinicas. Elderly individuals who agreed to take part by signing the Free and Informed Consent Form were included.

Besides the PAGes, from which some questions pertinent to the study objectives were selected, an analysis of 277 referral records involving other professionals of Hospital das Clinicas and respective reasons for these referrals was performed.

Lastly, interviews with sector heads were conducted using a questionnaire containing closed-ended questions. The questionnaire collected relevant data and information for subsequent use by residents as indicators of the main reasons for unplanned readmissions, repeat consultations and recurrences at the infirmary, outpatient clinic and day-hospital, respectively. These instruments and techniques were pivotal in the development of a Gerontology Care Plan for the services.

### Study investigation sites and criteria for using the service

Under the Brazilian National Health System (SUS), healthcare is structured into different levels of care, namely: primary care, medium complexity and high complexity. University teaching hospitals, such as Hospital das Clínicas of the Faculdade de Medicina of the Universidade de São Paulo (HC-FMUSP) of São Paulo State, are SUS-affiliated tertiary hospitals. These facilities engage in numerous activities such as primary health care promotion and prevention programs, secondary rehabilitation and prevention, primary care, social support, and homecare programs, among other specific care policies aimed at reducing hospital admissions.

Hospital das Clinicas is a leading healthcare, teaching and research institution and recognized center of excellence in the development of health technology. The hospital predominantly serves SUS users, but also delivers care to private patients holding health plans, given its extensive operating capacity, constant renewal of facilities and high-quality care staff.^7^

The Geriatrics Service of the HC-FMUSP boasts a multidisciplinary team specialized in treating elderly patients and is renowned for its educational training of geriatricians and gerontologists, as well as research studies conducted in the area of aging. The Geriatrics Service currently encompasses an Outpatient Clinic, Infirmary Ward and Day-care Hospital, as outlined below.

The Geriatrics Outpatient Clinic provides, on average, 11,900 consultations per year. The service operates on Wednesdays and Thursdays and deploys a team of 8 medical residents on each of these days. Users are referred to the Geriatrics Outpatient Clinic from the SG-HC Infirmary Ward, Day-care Hospital and other services, such as Interconsulta and the Hospital Auxiliar de Cotoxó.^7^ ^,^ ^8^

Information is recorded to guide diagnosis and treatment. These data can be collected using a variety of instruments, including: anthropometric measures, the Mini-Mental State Exam (MMSE), Geriatric Depression Scale (GDS), Tinetti Scale (POMA-Brasil), Katz Scale and Functional Independence Measure (FIM). Different members of the care team investigate aspects such as psychiatric disorders, gait disturbances and brief social issues.^7^ ^,^ ^8^

The Geriatric Infirmary of the HC-FMUSP is equipped with 17 beds and admits elderly patients aged ≥60 who present with different diseases not requiring surgical interventions or admission to an intensive care unit (ICU) at the time of hospitalization. In addition, patients whose therapeutic condition does not indicate palliative care are referred to this infirmary.^8^

Patients arrive at the Geriatric Infirmary from the Emergency Room, ICU, Geriatric Outpatient Clinic, or from other units in the hospital, such as homecare units. The multidisciplinary care team is made up of professionals trained in gerontology, including geriatricians and residents, nurses, speech-hearing and language therapists, psychologists, physiotherapists, nutritionists and social workers.

In 2007, the Geriatric Day Hospital (GDH) commenced operation with several objectives, including: to lower hospital admission/readmission rates of elderly; promote early discharge of elderly admitted to the infirmary; provide rapid resolution of problems diagnosed; establish a comprehensive diagnosis of patient health; carry out minor surgical procedures, offer guidance after hospital discharge and reduce costs related to patient management.^9^

The GDH boasts 18 beds and a team of geriatric physicians, a nurse, nursing assistants, a nutritionist and speech-language therapist.^9^

The criteria for referral to the GDH are: being aged >60; enrolled at the HC Complex or referred by the Paula Souza Health Center; presenting with acute decompensated refractory disease, infections, delirium or behavioral syndrome; requiring urgent diagnostic investigation, parenteral or intravenous drug infusion, treatment adherence control, certain procedures (tubes, tests, biopsies), blood transfusions or oral anticoagulation and functional rehabilitation.

At time of discharge, guidance is given by the team, and the patient is referred for the appropriate follow-up according to their needs.^9^ The aging process is accompanied by biopsychosocial aspects, calling for multidisciplinary care team qualified in catering to the wide variety of profiles inherent to the elderly population.^10^ A multidisciplinary team is therefore essential to enable implementation of actions that support the patient, caregiver and family.

Gerontology care should embrace components involving the assessment of psychosocial aspects and patient health status; care planning; solution coordination and implementation; treatment plan monitoring; and outcome assessment.^11^ Further, treatment non-adherence, as well as the culture, beliefs, values, economic conditions and family structure of patients should be taken into account.

Case management encompasses continuous patient-centered integral care which may be carried out by a professional or health team. Also, management is a cooperative process that diagnoses, plans, implements, coordinates, monitors and assesses options and services according to the patient’s health needs, through the available resources and communication.^12^

To this end, gerontology specialists, who hold multidisciplinary knowledge on the aging process, draw on their abilities and competencies to integrate and coordinate teams working with the elderly, performing case management to improve self-care, reduce care fragmentation, increase satisfaction of patient and of committed professionals, and make optimal use of the resources available.

According to Filho,^9^ on the abstract level, a gerontologist knows less about medicine and healthcare than a physician or nurse, less about psychology than a psychologist, and less about sociology than a sociologist or social worker. Nevertheless, again on the abstract level, this practitioner is more skilled than any of the specialists cited at developing and implementing activities involving the elderly and aging, from a holistic perspective of the life cyle.^10^ ^–^ ^12^

Lastly, gerontologists can help integrate professionals involved in patient care, establishing a focused consultation that is more cost-effective for patients, family and the institution.^13^ In this context, the study aim was to devise a gerontology management plan for both intramural and extramural use to determine the causes of readmission to the Infirmary, recurrence at the Day-Hospital and repeat consultation at the Outpatient Clinic. The study also sought to analyze the reasons for clinical referrals to other professionals in the hospital, to determine social and family profiles and screen risks and to analyze the functional capacity of patients interviewed.

## RESULTS AND DISCUSSION

Data collected using PAGe revealed that 50% of subjects were 71–80 years old and 33% >80 years, typifying the 21st century pattern of a rapidly growing contingent of older elderly (aged >80 years), a group more prone to frailty and more likely to depend on a support network for assistance in everyday activities;^14^ 42% were married and 42% widowed. The vast majority of the sample (67%) were women, confirming the phenomenon of feminization in old age, whereby females far outnumber males in the elderly population.^15^ With regard to education, 100% of the individuals reported incomplete primary education.

Results for the PAGe question on Autonomy and Functioning showed that 90% of elderly were independent for basic activities and 72% for instrumental activities, indicating that most were able to perform basic and instrumental activities of daily living without assistance, such as: domestic chores, feeding, dressing, etc.

With regard to Social and Family Conditions, 42% lived alone and 33% lived with others or spouse, while 25% lived with their son/daughter.

For self-perceived health, 25% of the elderly rated their health as very good, 33% as good, 29% as regular, and 12% as poor. None of the elderly classified their health as excellent. Overall, 67% of the sample reported having sought a physician more than twice in the past 12 months.

Another point investigated by the team of residents was an analysis of referrals made to other professionals following a medical consultation. The referral sheets (used by physicians from the outpatient clinic to request an assessment by other professionals on the team) are known internally as “Yellow Sheets” and contain requests normally filled out by the attending physician. A total of 277 Yellow Sheets were analyzed, predominantly involving referrals to social services (53%) and the psychology areas. The various professionals receiving the remaining 9% of referrals were: Physiotherapy, Urology, Occupational Therapy, Nutritionist, Physical Education specialist and Long-stay hospital.

The reasons underlying the most common referrals to the social service area were: social support network (lack of companion or caregiver), 53%; family problems, 20%; non-adherence to treatment, 12%; and medication-related problems, 10%. There were many reasons underlying treatment non-adherence or medication-related problems, including biological, psychological or social factors, such as fear of the disease, financial problems, educational and cultural level, forgetfulness, and self-medication.^14^ ^,^ ^16^ ^,^ ^17^ Other less frequent reasons included search for 3^rd^ age group, social benefits and equipment, among others.

The most important exponents of the elderly’s relationship network are family, friends and community. However, forms of family support have shifted with a major decline in birthrate (currently averaging 2.1 children born per woman), migration of young adults to more promising regions leaving their parents with no family support, and a growing number of women joining the workforce, factors that have reduced or eliminated female figures in the role of sole caregiver of dependent family members.^14^ ^,^ ^16^ In the area of psychology, 82% of referrals were due to the need for psychological support (psychotherapy, family problems, depression and grieving).

None of these yellow forms hold information on the outcome of the intra- or extramural referrals.

The most common reasons for referral to the Day-Hospital were uncompensated chronic diseases (47.6%) and infections (37.9%). Elderly often present with a number of chronic health problems such as arterial hypertension, arthropathies, diabetes and dementia, which can limit their everyday life.^18^ ^,^ ^19^ Similarly, infections are among the leading causes of hospitalization and death in the elderly. One of the main issues surrounding infections in this group is late diagnosis and consequent delay in treatment.^20^

Data collected from the interviews of sector heads revealed the absence of any formal recording of recurrences, return visits and readmissions or of missed routine outpatient consultations. Collecting this information would require individually searching all medical records.

The findings to date highlight the importance of integrating multidisciplinary information from medical charts and on treatment plans adopted to enable more effective management within the scope and situation of each patient, thereby promoting improved quality of life.

An integrated care approach can reduce the bureaucracy inherent to the service, rendering it more dynamic and less repetitive, while optimizing the use of human and material resources.

This effort is justified because it can deliver more comprehensive gerontology care, besides integrating the actions prescribed in multidisciplinary consultations via an integrating record. The approach also allows extramural management of cases with monitoring of self-care and resources needed, such as social, financial and psychological support. This management ensures prescriptions are followed, thereby preventing unplanned readmissions, consultations or recurrences. All instruments outlined below, when used in conjunction, provide integral complete gerontology management for patients and their relatives (micromanagement).

### Integrating record for case management

The first part of the proposal developed consists of an instrument that integrates measures taken by the multidisciplinary team, called the Integrating Record. The instrument comprises the following fields: service area (medicine, psychology, nursing and others), patient clinical status, care plan, objective, observations and attending physician. All these fields are completed by the gerontologist using information drawn from the patient’s medical record and are updated if and when the case requires.

**Figure 1:**
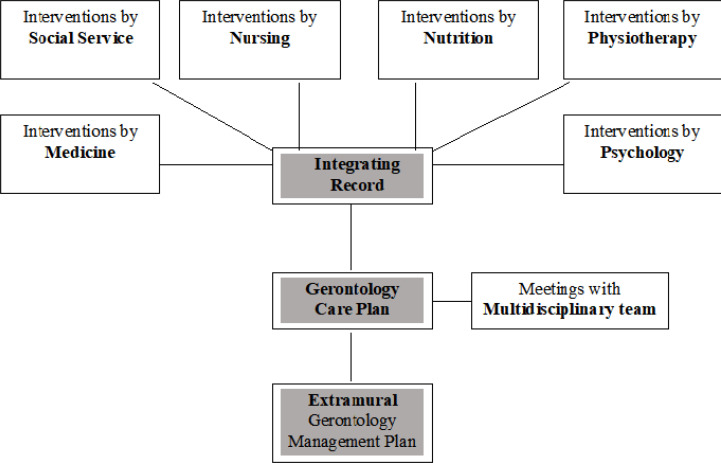
Integrating Record of Care interventions and referrals to Care Management for Elderly within Geriatric Services of a Tertiary Hospital, São Paulo, 2020.

The Integrating Record (depicted in Figure 1 and in Appendix A) is kept by the gerontologist, but any professional can access it or request information. Upon patient discharge, the Integrating Record is attached to the patient’s medical chart. The aim of the Record is to provide the gerontologist with sufficient information to devise a Gerontology Care Plan integrating the actions recommended by the other professionals, making these as pertinent as possible to the patient.

The Gerontology Care Plan is made up of four fundamental elements: a) planning and assessment of care; b) coordination and implementation of solutions; c) monitoring of care plan; and d) assessment of results. The plan also incorporates active ongoing reassessment allowing goals to be adjusted. Akin to the Integrating Record, the care plan should also be attached to the patient’s medical record.

To consolidate referrals and recommendations for gerontology care plans, gerontologists shall hold meetings with the team involved in treatment, providing them with the latest information on the patient’s compliance with actions, guidance, and prescriptions given. On the basis of this integrated data, the team can devise new strategies. Subsequently, these strategies will, after group consensus, be proposed to the patient and monitored by the gerontologist, consolidating the interface of the extramural and intrahospital care which are pillars of this management, as outlined below.

#### Extramural care plan, based on the integrating instrument

**Figure 2:**
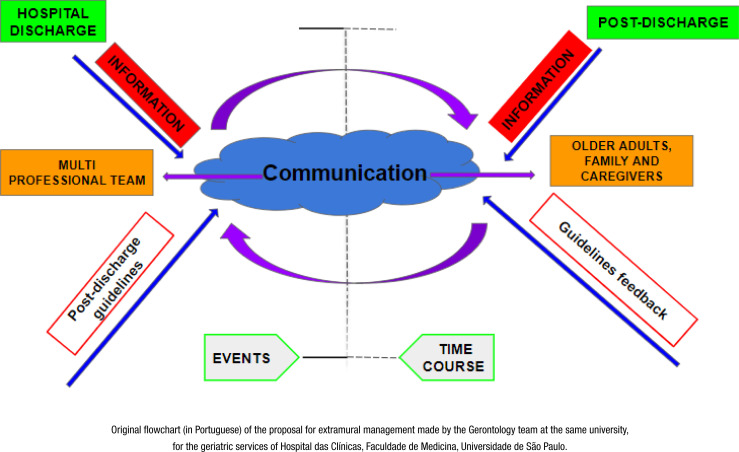
Flow diagram of extramural management, São Paulo, 2020.

Based on the Integrating Record and Gerontology Care Plan, the gerontology resident provides cases with extramural care, follow-up and monitoring of self-care and the necessary resources, such as social, financial and psychological support (Figures 1 and 2).

The monitoring is carried out by the gerontologist responsible for the case by means of regular telephone contact with patients and/or caregivers. Home visits can also be arranged according to the needs observed during follow-up. The purpose of this extramural care is to intensify monitoring of the plan, ensure prescriptions are followed, identify potential risks and provide guidance on the risks identified, thereby preventing unplanned readmissions, repeat consultations and recurrences.

Case management to optimize the interventions provided by the multidisciplinary care team in older adult patients is a care model that when employed as part of the healthcare action plan, transforms single-faceted care into multi-faceted care; that is, it encompasses biological, psychological, social and environmental needs, beyond the remit of traditional medical services.^21^ ^,^ ^22^

The role of the case manager (CM)^23^ includes identifying current and future needs of his/her client/patient, bringing together and coordinating the services available, advising patients and relatives, besides guiding and assisting them as service users. The CM scrutinizes the case, seeking all information necessary to devise a treatment plan. Subsequently, the CM implements and closely monitors this plan for effectiveness, adapting it to meet new needs, if and when they arise.

The term case manager is a derivative of case management, defined as a global, multidisciplinary effort centered on the individual and drawing on different areas of knowledge that address the needs detected. Heber^24^ points out that many professionals involved in case management tend to seek and explore only those needs related to their own area of expertise and background. The author states that there are at least four main case management models: Nursing, entailing managing health/disease, dysfunction and rehabilitation; Social Work, encompassing a range of social activities and variables; and Health Care, addressing health-related needs.

Another important aspect for case management is that care interventions of a generalist targeting health status can be run, constituting a key tool for promoting treatment adherence through education of the patient on their health condition and drugs treatment. Indeed, the literature indicates, as corroborated by the present study, there is great value associated with the knowledge held by the elderly on concepts such as health.^25^

Holding knowledge on how to cope and live daily with a chronic disease, such as hypertension, constitutes a valuable tool for implementing strategies that render treatment more effective and thus better manage the patient’s clinical condition.

In the context of the structural shift in contemporary society, i.e., a growing elderly population, it is clear that, besides the importance of specialized training of professionals qualified to cater to the health needs of this group, the available resources in the tertiary health service must be optimized to deliver this care effectively.

In the present study, there was an evident need for a gerontology management plan within the Geriatric Services of Hospital das Clinicas. The purpose of the management plan is to integrate actions performed by the multidisciplinary team through use of the Integrating Record. This instrument enables gerontologists to follow, propose a care plan and monitor cases, also extramurally, assessing the quality of social support networks and resources the elderly patient and family have at their disposal. It is noteworthy that this model of integrating record could potentially be applied to other medical areas and sectors besides geriatrics.

Case management can be applied in interventions to promote health in the elderly, prevent the collateral effects of comorbidities which may arise, assign new meaning to the ageing process and old age, expand the social support network, and provide guidance thereby increasing uptake of the use of services and promoting quality of life.

However, it is important to emphasize that case management, to be well planned and effective, requires a thorough multidimensional assessment of the elderly patient. Currently, several types of multidimensional assessment are typically used by all members of the multidisciplinary team, also referred to as comprehensive geriatric assessment (CGA).

The Gerontology Management Plan is broad and designed to prevent unplanned readmissions, consultations and recurrences as a result of poor adherence to interventions prescribed by professionals or of exposure to risk factors. As outlined earlier, the interviews conducted with heads of the sectors failed to elucidate the reasons for recurrences, repeat consultations or readmissions. Hence, given the absence of formal statistical records, this information would need to be gathered from all patient medical charts, requiring an extensive period of time.

Future studies should be conducted that enable ongoing assessment and refinement of the management plan goals proposed by the gerontology specialist at all stages of care: Integrating Record, Gerontology Care Plan and Extramural Gerontology Management Plan.

## Appendix A

**Table:** Integrating record of interventions by multidisciplinary team.

Date				
Professional/Name				
Action				
Strategy				
Results				
Facilitators				
Barriers				
Referrals				
